# A questionnaire to measure the quality of life in a forensic hospital

**DOI:** 10.1192/j.eurpsy.2021.1008

**Published:** 2021-08-13

**Authors:** M. Büsselmann, J. Streb, M. Dudeck

**Affiliations:** Department Of Forensic Psychiatry And Psychotherapy Günzburg, Ulm University, Günzburg, Germany

**Keywords:** Forensic, quality of life

## Abstract

**Introduction:**

Quality of life should be an important issue to everyone. In the general population and in various medical disciplines, this topic has been studied extensively. In forensic psychiatry, this topic has received little attention so far.

**Objectives:**

Within this project a questionnaire that measures the quality of life in forensic hospitals was developed.

**Methods:**

As a basis the questionnaire measuring the quality of prison life (MQPL) by Liebling et al. (2011), which was designed only for using in prisons, was used. First, this questionnaire was translated, then adapted to the living conditions in forensic hospitals and supplemented by questions regarding therapeutic support. For the psychometric evaluation of the instrument, a one-time survey was conducted at 13 forensic hospitals in Bavaria. A total of 255 patients took part, 25 of whom were female. In summary, the reliability of the MQPL can be rated as very good.

**Results:**

The factor structure was checked with a confirmatory factor analysis and was confirmed. There are significant differences between the 13 Bavarian forensic hospitals in the subscales admission to a forensic hospital, equal treatment, quality of accommodation and therapeutic services.

**Conclusions:**

The great importance of the quality of life in the penal system is shown by the fact that a good quality of life contributes to reduced psychological distress of inpatients.

